# Reply to Wang: Improving large language model approaches for identifying social determinants of health from clinical notes

**DOI:** 10.1073/pnas.2503187122

**Published:** 2025-03-20

**Authors:** Rodney A. Gabriel

**Affiliations:** ^a^Division of Perioperative Informatics, Department of Anesthesiology, University of California, San Diego, La Jolla, CA 92037; ^b^Department of Biomedical Informatics, University of California, San Diego Health, La Jolla, CA 92037

We thank Wang for their letter (1) regarding our publication on large language model (LLM)-based classifiers for identifying social determinants of health (SDoH) from electronic health record clinical notes (2). Wang points out the potential superiority of using LLM prompting to identify SDoH and the limitations of using synthetic data for training.

First, we agree that the use of zero- to few-shot prompting for SDoH classification tasks may exceed the performance as described. However, many LLMs are not pretrained on text from electronic health records due to privacy restrictions; thus, it may not directly capture the writing styles of clinicians. This has been shown to be a limitation for classification tasks in the clinical domain (3). Furthermore, clinical writing styles may differ across healthcare systems and thus, LLMs pretrained on clinical data from single institutions (such as ClinicalBERT) may still not generalize when tested on notes from separate institutions (4, 5). Future studies will need to focus on pretraining LLMs with clinical notes from diverse healthcare institutions to then evaluate LLM-prompting approaches for SDoH classification.

Wang cites references regarding the limitations of leveraging synthetic data, which report that the use of data augmentation strategies to generate training data did not improve transformer-based classification tasks (6) and that LLM-generated synthetic notes for training data did not improve classification tasks for problems with high *subjectivity* (7). We would like to highlight a few points regarding our approach:

(1) Data augmentation strategies, such as back translation, may not produce as diverse training data as LLM prompting. Diversity in training data may positively affect downstream performances of LLM-based models (8). Thus, the findings of the cited paper (6) may not necessarily be generalizable to the approach used in our study.
(2) In another study cited by Wang (7), the authors demonstrated that synthetic data generated from zero- and few-shot prompting did not improve classification tasks with labels that had high *subjectivity*. In contrast, labeling of SDoH may arguably be more *objective* than subjective (e.g., a patient is homeless, a patient experiences domestic violence). Indeed, in that cited study, (7) models trained on synthetic data versus real-world data performed more similarly to each other for objective compared to subjective classification tasks.
(3) We took the approach of combining synthetic training data with real-world data, which demonstrated improvement in accuracy as well as generalizability. However, we did not perform an experiment on measuring a ceiling effect on the quantity or proportion of synthetic data in training data on model performance. Such an experiment is indeed important.

Exploration of the use of prompting LLMs that are pretrained with clinical data from diverse sources is essential. Due to the limitation with sharing clinical data across diverse sites, the use of synthetic data may be beneficial in the clinical domain especially when labeled datasets are limited. However, as Wang opines, there are key potential limitations of synthetic data for LLM-based classifiers that need to be further defined.
